# Fully *in situ* Nb/InAs-nanowire Josephson junctions by selective-area growth and shadow evaporation†

**DOI:** 10.1039/d0na00999g

**Published:** 2021-01-19

**Authors:** Pujitha Perla, H. Aruni Fonseka, Patrick Zellekens, Russell Deacon, Yisong Han, Jonas Kölzer, Timm Mörstedt, Benjamin Bennemann, Abbas Espiari, Koji Ishibashi, Detlev Grützmacher, Ana M. Sanchez, Mihail Ion Lepsa, Thomas Schäpers

**Affiliations:** Peter Grünberg Institut (PGI-9), Forschungszentrum Jülich 52425 Jülich Germany th.schaepers@fz-juelich.de +49 2461 61 2940 +49 2461 61 2668; JARA-Fundamentals of Future Information Technology, Jülich-Aachen Research Alliance, Forschungszentrum Jülich, RWTH Aachen University Germany; Department of Physics, University of Warwick Coventry CV4 7AL UK; RIKEN Center for Emergent Matter Science and Advanced Device Laboratory 351-0198 Saitama Japan; Peter Grünberg Institut (PGI-10), Forschungszentrum Jülich 52425 Jülich Germany

## Abstract

Josephson junctions based on InAs semiconducting nanowires and Nb superconducting electrodes are fabricated *in situ* by a special shadow evaporation scheme for the superconductor electrode. Compared to other metallic superconductors such as Al, Nb has the advantage of a larger superconducting gap which allows operation at higher temperatures and magnetic fields. Our junctions are fabricated by shadow evaporation of Nb on pairs of InAs nanowires grown selectively on two adjacent tilted Si (111) facets and crossing each other at a small distance. The upper wire relative to the deposition source acts as a shadow mask determining the gap of the superconducting electrodes on the lower nanowire. Electron microscopy measurements show that the fully *in situ* fabrication method gives a clean InAs/Nb interface. A clear Josephson supercurrent is observed in the current–voltage characteristics, which can be controlled by a bottom gate. The large excess current indicates a high junction transparency. Under microwave radiation, pronounced integer Shapiro steps are observed suggesting a sinusoidal current–phase relation. Owing to the large critical field of Nb, the Josephson supercurrent can be maintained to magnetic fields exceeding 1 T. Our results show that *in situ* prepared Nb/InAs nanowire contacts are very interesting candidates for superconducting quantum circuits requiring large magnetic fields.

## Abstract 2

Nb/InAs-nanowire Josephson junctions are fabricated *in situ* by a special shadow evaporation scheme for the superconducting Nb electrode. The junctions are interesting candidates for superconducting quantum circuits requiring large magnetic fields.

## Graphical abstract

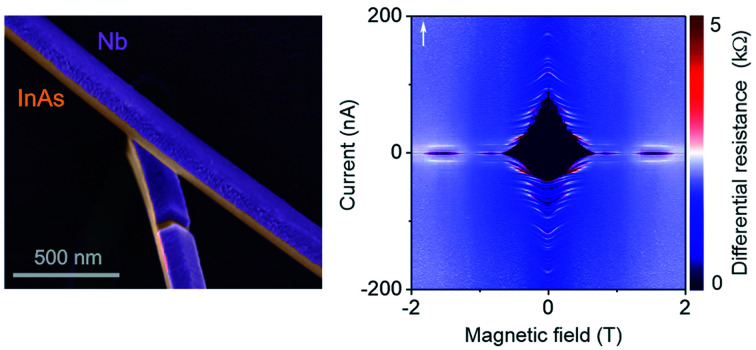

## 1 Introduction

III–V semiconductor nanowires combined with superconducting electrodes are versatile building blocks for various applications in the field of quantum computation and experiments addressing fundamental aspects of quantum nanostructures. Josephson junctions formed by two superconducting electrodes bridged by a nanowire segment allows the control of the critical current by a gate voltage.^1–3^ Such an approach, results in a much more compact superconducting circuit lay-out compared to the common flux-controlled one. This advantage is used *e.g.* in a gatemon qubit, a special form of the transmon qubit, in which the Josephson junction in the qubit resonator circuit is controlled by a gate.^4–7^ Furthermore, owing to the large Fermi wavelength of the electrons in the semiconductor combined with the small diameter of the nanowire, a finite number of discrete Andreev bound states that carry the Josephson supercurrent are formed. Coherent transitions between these discrete states can be used in Andreev level qubits for quantum circuits.^8–10^ Apart from these more conventional qubit applications, nanowire–superconductor hybrids are also very promising for topological qubits based on Majorana fermions.^11–15^

InAs and InSb are the common semiconductors of choice to form a highly transparent interface with the superconducting electrodes, a prerequisite for a sufficiently large supercurrent. Depending on the application, different superconductor materials are deposited. Thus, for example, aluminium with a small superconducting gap and a small critical magnetic field but large superconducting coherence length is a common choice.^1,13,16^ For higher temperatures or higher magnetic fields operation, superconductors such as Nb and its alloys,^3,15,17,18^ Pb,^19,20^ or V,^21,22^ are used. However, detailed analysis of these semiconductor/superconductor systems are just starting to emerge.

Until recently, the conventional methods to produce superconductor–semiconductor interfaces, are based on the semiconductor surface cleaning (by wet chemical etching or Ar^+^ sputtering) prior to the superconductor deposition.^17^ The major issues with these *ex situ* approaches are the presence of residual atoms and the semiconductor surface damage that results in a non-ideal interface. Consequently, a soft induced gap might form in the semiconductor nanowire with a significant density of states present within the superconducting gap induced by the proximity effect.^17^ This effect is especially detrimental for topological qubits based on Majorana fermions. A successful method to circumvent these problems is the *in situ* deposition of the superconducting material on the semiconductor nanowires.^18,22–24^

Another important issue is the residual material left on the structure from wet-chemical etching technique, that is normally used for the fabrication of Josephson junctions with a small gap. However, recently it was demonstrated that closely separated superconducting electrodes can also be achieved by shadow evaporation technique, *i.e.* either by using a nanowire which crosses another^25–27^ or using a patterned, suspended SiO_2_ layer as a shadow or stencil mask.^18,22^ All of these methods, have a common trait to potentially reach the so-called short channel limit, in which the current through the device is only carried by a single Andreev bound state. Such mesoscopic junctions are one of the main requirements for topological systems based on Majorana fermions. Additionally, the lack of higher levels, suppresses possible loss channels for the Andreev qubit.

Here, we combine both of the above approaches to report the fabrication of a fully *in situ* Josephson junction, based on the evaporation of superconductor half-shells on InAs nanowires by a specially designed shadow evaporation technique. Pairs of NWs are selectively grown on different Si (111) adjacent facets, in a way that one NW shadows the other one situated at a small distance. Thus, the deposited junction width is determined by the nanowire diameter and the distance between them. Although our approach is applicable to almost any superconductor material, here we focussed on Nb, since small separations between Nb superconducting electrodes are difficult to fabricate by other methodologies like buffered chemical polishing due to the aggressive chemical etchants, *e.g.* hydrofluoric (HF), nitric (HNO_3_), and phosphoric acid (H_3_PO_4_).^28^ The InAs/Nb interface of the Josephson junctions fabricated by our novel approach are analysed in depth by electron microscopy techniques. Low temperature transport properties of the Josephson junctions are also reported.

## 2 Results and discussion

### 2.1 *In situ* prepared InAs–Nb junctions

**Fig. 1:**
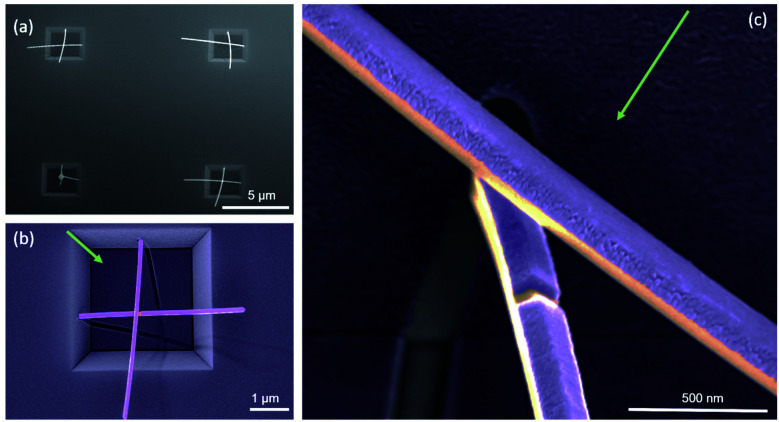
Scanning electron microscopy images of selectively grown nanowire junctions: (a) Overview of the 3 μm square troughs with selectively grown nanowires (top view). (b) False-coloured single square with two Nb covered nanowires grown off the adjacent Si (111) facets. The shadow depicts the direction of the metal (Nb) deposition (green arrow). (c) Close-up of the crossing section, showing the gap in the Nb layer (purple) in the bottom nanowire (orange) due to the shadowing from the top nanowire (30° titled image).

InAs-nanowire growth was carried out by molecular beam epitaxy (MBE). To enable selective area growth, 3 μm wide square-shaped troughs are etched on to Si (100) substrates using SiO_2_ as a mask. The resulted side facets of the troughs are Si (111) usually used for the growth of nanowires. After mask removal, the whole surface of the substrate is re-oxidized. Finally, nano-holes are etched in the new SiO_2_ on the side facets of the trough, defining the position of the nanowires. An offset of 100 nm from the center of the facet is imposed to enable nanowires from neighboring facets to cross each other closely without merging. The InAs nanowires are grown by self-assisted nucleation and vapour-solid (VS) mechanism.^29^ Following the growth of 4–5 μm long and 80 nm diameter InAs nanowires, the sample was transferred to a metal MBE chamber for the Nb half-shell deposition. A scanning electron microscopy (SEM) image with crossed nanowires are shown in Fig. 1a. A false-coloured magnified top-view image of one square trough is presented in Fig. 1b with two Nb-covered nanowires grown on adjacent Si (111) facets. The Nb gap on the bottom nanowire is clearly observed in the further magnified image in Fig. 1c, which confirms the formation of the Josephson junction with the separation of the two Nb electrodes defined by the shadow of the upper nanowire.

### 2.2 Transmission electron microscopy

**Fig. 2:**
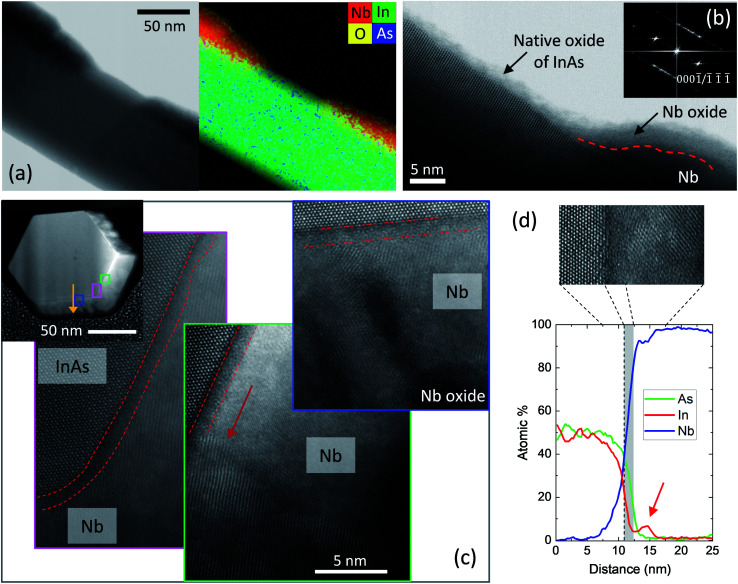
(a) Bright field (BF) and annular dark field (ADF) STEM images of a junction, with the EDX elemental maps superimposed on the ADF image. A Nb-free gap is revealed. (b) High magnification image of the gap region with the corresponding FFT (inset) (c) ADF image of a nanowire cross section and higher magnification images from the three regions indicated by squares. The amorphous layers are marked by the red broken lines in the higher magnification images. The red arrow points to a grain boundary. (d) EDX line profile along the yellow arrow shown in (c). The red arrow indicates the increase in In within the Nb layer and the greyed area marks the amorphous region shown in the inset high magnification ADF image.

Due to the shadowing from the closely placed thin upper nanowire, junctions with separations of few tens of nanometers can be formed. Fig. 2a shows a bright field scanning transmission electron microscope (STEM) image of a Nb–InAs junction and the corresponding energy dispersive X-ray (EDX) elemental map, superimposed on the annular dark field (ADF) image. The separation is ∼55 nm and the junction is clean, *i.e.* with no traces of Nb within the gap. Along rest of the nanowire, a uniform and continuous Nb layer is formed, with only ±2 nm variation in thickness along the nanowire. Fig. 2b shows a high magnification ADF image of the gap and the corresponding fast Fourier transform (FFT) inset. The polytypic crystal structure of the InAs nanowire can be seen in the gap region, and the ZB/WZ spots in the FFT confirms the same. More details on the crystal structures of InAs nanowires and the Nb shell can be found in Fig. S5a, b and d in ESI.† Since no Nb is detected within the gap by EDX measurements, the thin amorphous oxide layer on InAs weaklink segment Fig. 2b corresponds to the native oxide commonly formed on the InAs nanowires due exposure to air after growth of the nanowire Josephson junction. Fig. 2c shows an ADF image of a nanowire cross-section, along with three higher magnification images of the interface from the regions indicated. Nb is deposited on three of the six ({112̄0} type) side facets (see ESI Fig. S5b†), in agreement with the SEM observations in Fig. 1d. Deposition on the middle facet is thicker (∼22 nm), smooth and formed by relatively large grains of ∼15–30 nm (one grain boundary is indicated by a red arrow). In contrast, those on the two facets on the sides are column-like, poly-crystalline in structure and lower in thickness (∼16 nm). This is due to the difference between the effective deposition angles on different facets. The metal flux is almost perpendicular to the nanowire axis at 87°, and is directed at the middle facet. Which, also aided by the substrate temperature,^24^ results in a smooth growth on this facet. The effective angles created with the two facets on either side are steep with a smaller deposition angles, resulting in column-like growth.^30^

Closer inspection of the InAs–Nb interface reveals a ∼1 nm uniform amorphous layer on all three facets (marked by the red dashed lines). Fig. 2d shows an EDX line scan across the interface at the position indicated by the yellow arrow in Fig. 2c. Two interesting observations can be made in the composition variation from InAs to Nb at the interfaces of these nanowires. Firstly, it can be seen that the As composition remains relatively high within the first ∼1.5 nm following the interface (grey area in Fig. 2d), while In composition is much lower in this region. Secondly, In, which initially shows a dip within the amorphous region, slightly increases afterwards, before decreasing again (red arrow). The compositions of the amorphous layer measured across different facets and nanowires were found to vary between As: 25–40% In: 5–20% and, Nb: 45–60% (considering only Nb, As and In). One example is shown in ESI Fig. S5c.† Although the composition values of the 1 nm layer cannot be ascertained precisely due to the contributions from layers on either side, it is clear that this region contains a higher percentage of As and Nb. Considering the ternary phase diagram between In–As and Nb at room temperature,^31^ one could see that there is no tie-line between InAs and Nb. This means that InAs and Nb cannot exist in equilibrium. Instead, they react in a dominant reaction^31^ and form compounds, even at room temperature. The crystal structure of the layer formed by mixing (or solid diffusion) is amorphous, similar to many semiconductor–metal interfaces that show similar behaviour of solid state amorphisation.^15,32,33^

As the amorphous layer contains much less In than As, the excess In from InAs decomposition is expelled and segregated on the Nb side, forming an In rich band. The existence of tie-lines between number of Nb_*x*_As_*y*_ and In/Nb_3_In in the In–As–Nb phase-diagram,^31^ suggests that compounds of the former can co-exist with In or Nb_3_In. Similar observations have been made in other material systems such as Pt–GaAs, where gallide formation took place close to the metal interface and arsenide formation close to the semiconductor interface during re-crystallisation.^33^ Although the subsequent transport measurements do not indicate significant effects from this amorphous layer, the current results bring to notice the important aspect of room temperature reactions and amorphisation of semiconductor–superconductor/metal interfaces.

### 2.3 Electrical characterization

**Fig. 3:**
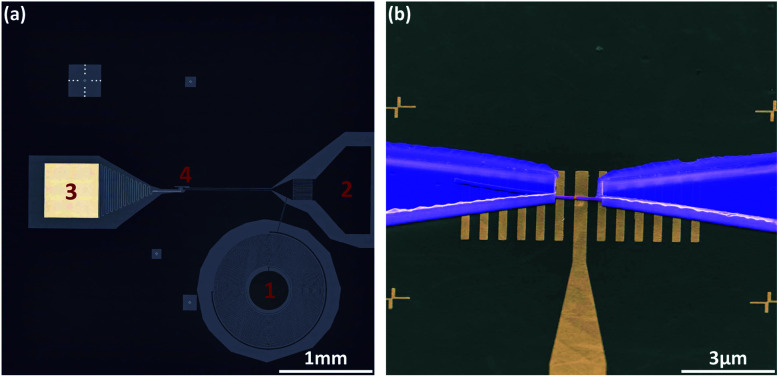
(a) Optical microscope image of a bias-tee chip implemented by combining a coil (1) and an inter-digital capacitor (2) connected to one side of the junction. The other side is connected to the global ground plane. For electrostatic tuning, we use a bottom gate electrode, which is terminated by a large bonding pad (3). The junction is located at (4). (b) Scanning electron micrograph of an InAs nanowire covered by Nb half-shells, which are contacted by NbTi fingers. The junction is placed on a bottom-gate electrode. The metal finger grid on either sides of the gate are for mechanical support of the nanowire.

**Fig. 4:**
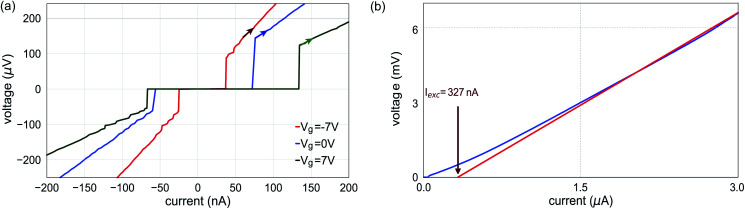
(a) Current–voltage characteristics of an InAs/Nb shadow junction measured at *V*_g_ = 0 V, −7 V, and 7 V, showing an almost three-fold reduction of the critical current between the largest and smallest gate voltages. The sweep direction is indicated by arrows. The junction is slightly underdamped and shows a small hysteretic behaviour due to overheating. (b) I–V characteristics of the same junction for large bias currents and a gate voltage of *V*_g_ = 7 V. Based on the measurement we obtain an excess current *I*_exc_ = 327 nA and a normal state resistance *R*_N_ = 2850 Ω.

The nanowire Josephson junction was integrated in an on-chip bias tee, containing an inter-digital capacitor and a planar coil, to perform both AC and DC measurements (*cf.*Fig. 3a). The Nb shells were contacted by NbTi fingers, while control of the carrier concentration of the InAs segment between the Nb electrodes was achieved by means of a bottom gate. A scanning electron micrograph of the junction device is depicted in Fig. 3b. In order to get an overview of the junction properties the current–voltage (I–V) characteristics at three different gate voltages at the temperature of 15 mK were measured. As can be seen in Fig. 4a, at zero gate voltage a relatively large switching current of *I*_c_ = 75 nA and a re-trapping current of *I*_r_ = 60 nA are observed, which is inline with previous studies on Nb/InAs nanowire junctions.^3^ For larger gate voltages, *i.e. V*_g_ = 7 V, those values increase to *I*_c_ = 133 nA and *I*_r_ = 67 nA, respectively. Whereas, for *V*_g_ = −7 V the switching and retrapping currents are lowered to values of about 40 nA and 25 nA, respectively. Thus, between the smallest and largest gate voltage the junction exhibited an almost tripling of the switching current. The fact that *I*_r_ is only slightly lower than *I*_c_ indicates that the heating effect caused by dissipation in the resistive state is only moderate. As a result of the special circuit geometry, namely the coupling to a coplanar waveguide transmission line, the junction is affected by the emission and self-absorption of photons due to the AC Josephson effect, resulting in so-called self-induced Shapiro steps on the re-trapping branch. The decrease *I*_c_ with decreasing *V*_g_ can be attributed to the fact that by lowering the carrier concentration the number of transport channels carrying supercurrent *via* phase-coherent Andreev reflections is reduced. However, for even more negative gate voltages, no complete suppression of *I*_c_ could be achieved. We attribute this to the incomplete pinch-off of the electron gas in the InAs nanowire bridge segment. This is in contrast to our Al-based nanowire junctions for which all transport could be completely suppressed.^34^ A possible reason is that the *in situ* deposited Nb layer changes the Fermi level pinning at the interface leading to an enhanced carrier accumulation at the interface. Furthermore, the structural properties, like the alloying and the amorphous layer at the interface may have an affect as well. As a consequence of the inability to pinch-off the junction, no tunnel spectroscopy could be performed, in order to find out about the hardness of the induced gap. However, due to the large superconducting gap of Nb, *e.g.* compared to the gap of Al, a more robust supercurrent is maintained in the junction.

In order to obtain information about the junction transparency, we measured the *I*–*V* characteristics up to large bias voltages at *V*_g_ = 0 V. By linear extrapolation in the bias voltage range above 2*Δ*/*e* we were able to extract an excess current of *I*_exc_ = 327 nA and a normal state resistance of *R*_N_ = 2850 Ω. By utilizing the framework of the corrected Octavio–Tinkham–Blonder–Klapwijk theory,^35,36^ we obtain a ratio of *eI*_exc_*R*_N_/*Δ* = 0.623 which results in a barrier strength of *Z* = 0.69. The latter corresponds to a transparency 
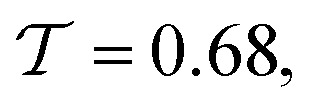
 which is a typical value for a nanowire Josephson junction with a wide-gap superconductor like Nb in the many channel regime.^3^ The junction transparency is large, with no significant detrimental effect apparent from the amorphous interfacial layer observed in the TEM studies.

**Fig. 5:**
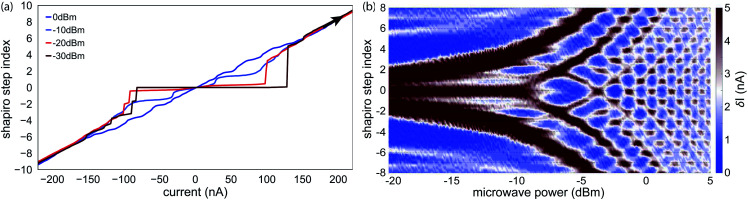
(a) Current–voltage traces for different microwave excitation powers at a fixed frequency of *f* = 5 GHz. While the trace at −30 dBm (dark red) reproduces the current–voltage curves without any additional AC component, the pronounced plateau region within the zero voltage state is replaced by equidistant voltage steps when the power is increased. The sweep direction for all measurements is indicated with the black arrow. (b) Histogram of the power-dependent Shapiro response for a constant microwave frequency of *f* = 4 GHz.

For mesoscopic Josephson junctions, the device response to an applied microwave signal provides information about the internal state structure, such as presence of a topological state.^37,38^ Thanks to the combination of the coplanar waveguide transmission line and the bias-tee, the nanowire Josephson junction can be supplied with an AC and DC signal simultaneously with efficient transmission to the junction over a wide range of frequency, which would be difficult to achieve with an external antenna. Fig. 5a shows a set of current–voltage traces for a fixed frequency of *f* = 5 GHz and different microwave powers at *V*_g_ = 7 V. For low power, *i.e.* −30 dBm, the curve mimics the behavior of a purely DC-driven junction. However, if the power is increased, the zero voltage state is gradually suppressed and replaced by equidistant voltage plateaus, so-called Shapiro steps, of height *n* × *hf*/2*e*, with *h* Plancks's constant and *n* = 1, 2, 3, …. Originating from the AC Josephson effect, they are tightly bound to the current–phase relation and damping behavior. However, while all integer steps are well pronounced, indicating a single well-defined junction, there is no obvious indication for any non-trivial features such as missing steps. Most importantly, there are no signs of subharmonic steps, which may be observed as a consequence of a non-sinusoidal current–phase-relationship due to a interface transparency close to unity.

As one can see in Fig. 5a, the quality and shape of the Shapiro steps also depend on the effective microwave power that is applied to the junction. Thus, we performed a more systematic mapping of the AC response for constant frequencies. Fig. 5b shows the histogram of binned voltage data scaled by the current step size of the measurement at *f* = 4 GHz for microwave powers at the input port of the fridge between −20 dBm and + 5 dBm. For low powers, the junction exhibits a chevron-like pattern without well-defined steps. The latter can probably be attributed to the fact that the small amplitude of the AC drive is not sufficiency to maintain a resonant motion of the particle in the washboard potential and resistively shunted junction (RSJ) model. However, when the microwave power is increased above −5 dBm, clearly pronounced Shapiro steps are observed.

**Fig. 6:**
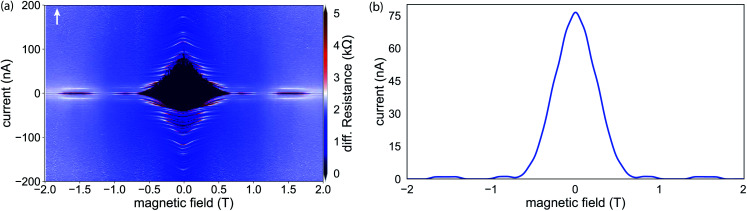
(a) Magnetic field dependent differential resistance for *V*_g_ = 0 V. The field is oriented in-plane along the nanowire axis. For both sweep directions, the nanowire junction exhibits a fluctuating resistance that corresponds to the alternating suppression and revival of the supercurrent. This effect can be attributed to a mixture of spin–orbit interaction and the interference between multiple transverse modes in the nanowire.^39^ The observed behavior is maintained for magnetic fields above 2 T, indicating a comparably large critical field *B*_c_. (b) Field-dependent magnitude of the switching current, clearly showing the reappearance of the supercurrent for fields up to 2 T.

Operation in the presence of a magnetic field is common to all structures that are based on few-channel mesoscopic nanowire Josephson junctions, *i.e.* the Andreev qubit or topological systems. In the case of the former, for example, the system can operate as intended if the junction and the connected superconducting loop is exposed to a magnetic flux *Φ* = *Φ*_0_/2 corresponding to a phase bias of π, with magnetic flux quantum *Φ*_0_ = *h*/2*e*. For the creation of Majorana zero modes, on the other hand, one needs a strong in-plane field that can easily exceed hundreds of milli-Tesla. This is especially true in the case of InAs due to the smaller *g*-factor if compared with InSb.^40^ Thus, the magnetic field robustness of the induced superconductivity in the nanowire junction is of special interest to benchmark the device performance. Fig. 6a shows the device response in terms of the change of the differential resistance, if the system is penetrated by a magnetic field in parallel to the nanowire axis. Here, the most obvious feature is the lobe-like pattern centered around zero magnetic field. Considering the center lobe, the supercurrent is suppressed at large magnetic field magnitudes of around ±0.7 T. The strong asymmetry along the current axis close to zero magnetic field can be attributed to the difference of the switching and retrapping current. The stripe-like structure in the retrapping branch is due to the existence of self-induced Shapiro steps. We find that the field-dependency of the supercurrent does not follow the expected monotonous decrease when the superconducting gap energy decreases with the magnetic field. Instead, the device exhibits an alternating, non-periodic series of sections with and without a supercurrent. These lobe-like structures at higher magnetic fields, in which the supercurrent reappears for a finite field range, can probably be attributed to the intermixing and interference of multiple but not-too-many transverse modes.^39^ The corresponding, field-dependent magnitude of the switching current depicted in Fig. 6b clearly shows that the lobe-structures do not follow a typical Fraunhofer-like pattern and the junction can still host finite supercurrent even up to 2 T.

## 3 Conclusion

Our results show that highly transparent Josephson junctions can be fabricated by combining selective-area growth with a shadow evaporation scheme for the superconducting electrodes. The transmission electron microscopy investigations confirmed the absence of any foreign residue at the interface between the superconductor and the InAs nanowire. However, a very thin amorphous layer is observed at the interface. The Nb growth on the middle facet is found to be smooth consisting of large grains, while the side facets are polycrystalline and column-like. Owing to the large interface transparency, the junctions showed a clear signature of a Josephson supercurrent in the transport experiments. Gate control was possible, however, compared to Al-based junctions prepared in a similar fashion no complete pinch-off was achieved, which may be due to an enhanced surface accumulation in InAs when in contact to Nb. The Shapiro steps observed in the *I*–*V* characteristics show pronounced integer steps, indicating a sinusoidal current–phase relation. Taking the magnetic interference effects into account we have strongly evidenced successful fabrication of a weak link which works well even in high magnetic fields.

Our Nb/InAs nanowire-based junctions prove to be very interesting devices, with great potential for applications in superconducting quantum circuits that require large magnetic fields. In fact, most of the advanced approaches for the detection, manipulation and utilization of topological excitations, like the Majorana-transmon, rely on phase-sensitive and well-controlled detector structures. Here, our InAs/Nb *in situ* nanowire shadow Josephson junctions can act as ideal gate-tunable components in superconducting quantum interference device (SQUID) structures which do not exhibit pronounced quantum fluctuations of the supercurrent and, additionally, maintain their superconducting properties even at large magnetic fields. The exact origin of the non-ideal transparency remains unclear. Even though we do not see any obvious indications in our measurements that the transport properties are altered by the interlayer, it still interesting for its effects to be investigated experimentally. Further improvement of the nanowire Josephson junctions may be possible by post-growth annealing and hence inducing re-crystallisation of the amorphous layer at the Nb/InAs interface. Additionally, an improvement of the general crystal quality is desirable, as the combination of the small mean free path,^41,42^ which has been measured for other self-assisted nucleation grown nanowires, and the small coherence length of Nb clearly put our Josephson junctions into the diffusive regime. Under this condition, we expect no direct relation between the individual number of defects within a specific Nb/InAs nanowire junction and its device response, *e.g.* the supercurrent.^41^ However, the comparably large defect density might cause some obstacles for future qubit applications. Especially parasitic two-level systems could interfere with the cavity-qubit coupling, induce additional loss channels and thereby limit the lifetime of the whole system.

## 4 Methods

### 4.1 Substrate preparation and nanowire growth

The pre-pattered Si substrates used for the selective-area growth of the InAs nanowires were prepared by using a three-step electron beam (e-beam) lithography fabrication process, *i.e.* the first step places the alignment markers for the last one, the second defines the square-shaped troughs, and the third step is employed to define pairs of nano-holes on adjacent side facets of the square troughs. The substrates used for the template fabrication are Si (100) wafers, thermally oxidized to get 20 nm of SiO_2_. The alignment markers necessary in the third e-beam lithography step are defined by reactive ion etching (RIE). Next, arrays of 3 μm wide square troughs with a pitch of 10 μm are etched in the Si (100) substrate (*cf.* Fig. S1a in ESI†). Here, patterned PMMA resist is used as a mask to anisotropically etch the SiO_2_ layer by RIE using CHF_3_ and oxygen until the Si (100) surface is revealed. Subsequently, the resist mask is removed and the Si (100) is etched for 90 s with tetramethyl ammonium hydroxide (TMAH) to obtain the 300 nm deep square-shaped troughs with Si (111) facets (*cf.* Fig. S1b†). Next, the oxide on the substrate surface is removed completely with buffered HF. Subsequently, a thermal re-oxidation is performed resulting in a 25 and 18 nm thick oxide layer on the (111) and (100) surfaces, respectively (*cf.* Fig. S2†). As a part of the third lithography step, 80 nm wide holes are defined in the oxide layer on the side facets for the subsequent selective-area growth. The holes are etched using a combination of RIE and HF wet chemical etching (*cf.* Fig. S1c†). A focused ion beam etching prepared cross-sectional cut of a 80 nm wide hole on a Si (111) facet is depicted in Fig. S1d.† Regarding the position of the holes an offset of 100 nm from the center of the facet is imposed to enable nanowires from neighboring facets to cross each other closely rather than merging into one crystal.

The InAs nanowires are selectively grown in the holes on the Si (111) facets *via* molecular beam epitaxy (MBE). A vapour-solid method without any catalyst is employed. In the first step the nanowires are grown at a substrate temperature of 480 °C with an indium growth rate of 0.08 μm h^−1^ and an As_4_ beam equivalent pressure (BEP) of ≈4 × 10^−5^ mbar for 10 min to sustain an optimal growth window and then in the second step the substrate temperature is decreased to 460 °C with an indium growth rate of 0.03 μm h^−1^ and As_4_ BEP of ≈3 × 10^−5^ mbar for 2.5 h resulting in 4–5 μm long and 80 nm wide nanowires.

After the growth of the InAs nanowire, the substrate undergoes arsenic desorption at 400 °C for 20 min and at 450 °C for 5 min. Subsequently, the sample is transferred to a metal MBE chamber. Further on, the Nb metal shell is deposited at an angle of 87° to the nanowire axis. The Nb is evaporated at a substrate temperature of 50 °C. The measured growth rate of 0.082 nm s^−1^ resulted in an average Nb thickness on the nanowire of 17 nm.

Further processing details on substrate preparation and nanowire growth can be found in the ESI.† In addition, as elaborated also in the ESI,†*in situ* InAs nanowire-based Josephson junctions can also be fabricated by using random nanowire growth on adjacent Si (111) side facets. This approach offers the advantage of easier fabrication but has the disadvantage of uncontrolled junction formation (*cf.* Fig. S4†).

### 4.2 Device fabrication

The devices for electrical characterization were fabricated on highly-resistive Si substrates with pre-patterned bottom gate structures and a superconducting circuit, as shown in Fig. 3a. As the devices are intended to work for both AC and DC measurements, we use a transmission line in coplanar waveguide geometry to form the source contact of the nanowire Josephson junction. The latter is terminated by an on-chip bias tee, consisting of an inter-digital capacitor and a planar coil. All three elements, together with the surrounding ground plane, were made of reactively sputtered TiN with a thickness of 80 nm. Subsequently, the nanowires were deposited onto the electrostatic gates by means of a SEM-based micro-manipulator setup. To ensure an ohmic coupling between the contacts, made of NbTi, and the Nb shell, we used an *in situ* Ar^+^ dry etching step prior to the metal deposition. The contact separation is chosen to be at least 1.5 μm in order to reduce the effect of the wide-gap superconductor NbTi on the actual junction characteristics. The finished junction device is depicted in Fig. 3b.

### 4.3 Transmission electron microscopy

For the side view analysis, the nanowires were transferred from growth arrays to holey carbon grids by gently rubbing the two surfaces. The cross sections were prepared using focused ion beam (FIB) on nanowires transferred to Si substrates by the same method of gentle rubbing of surfaces. TEM analysis was carried out using doubly corrected Jeol ARM 200F and Jeol 2100 microscopes, both operating at 200 kV. The EDX measurements were carried out using an Oxford Instruments 100 mm^2^ windowless detector installed within the Jeol ARM 200F.

### 4.4 Electrical measurements

The electrical measurements were performed in a ^3^He/^4^He dilution refrigerator with a base temperature of 13 mK. The current–voltage characteristics were measured in a quasi four-terminal configuration using a current bias. For the differential resistance measurements a standard lock-in technique was employed. The rf-frequency signal for the measurements of the Shapiro steps was applied to the junction *via* the capacitor of the bias-tee.

## Author's contribution

P. P., T. M., P. Z., J. K., B. B., A. E. and M. I. L. performed the nanowire growth by MBE and fabricated the samples for transport experiments and carried out the basic electrical characterization, H. A. F., Y. H. and A. M. S. conducted to the structural analysis by TEM, P. Z. and R. D. performed the low-temperature transport experiments. P. P., H. A. F., P. Z., R. D., A. M. S., M. I. L. and T. S. wrote the manuscript. All authors contributed to the discussions.

## Conflicts of interest

There are no conflicts to declare.

## Supplementary Material

NA-003-D0NA00999G-s001
