# Supplementary material for: Fully in situ Nb/InAs-nanowire Josephson junctions by selective-area growth and shadow evaporation
Source: Nanoscale Adv. 2021 Jan 19;3(5):1413–21. doi: 10.1039/d0na00999g (PMC9418346; doi:10.1039/d0na00999g)
Supplement: NA-003-D0NA00999G-s001 [file NA-003-D0NA00999G-s001.pdf]

Electronic Supplementary Material (ESI) for Nanoscale Advances.
This journal is © The Royal Society of Chemistry 2021

Supporting Information: Fully in-situ Nb/InAs-nanowire Josephson junctions by selective-area growth and shadow evaporation.

Pujitha Perla,[1,2] H. Aruni Fonseka,[3] Patrick Zellekens,[1,2] Russell Deacon,[4] Yisong Han,[3] Jonas Kölzer,[1,2] Timm Mörstedt,[1,2] Benjamin Bennemann,[1,2] Abbas Espiari,[1,2] Koji Ishibashi,[4] Detlev Grützmacher,[1,5,2] Ana M. Sanchez,[3] Mihail Ion Lepsa,[5,2] and Thomas Schäpers[1,2]

[1]*Peter Grünberg Institut (PGI-9), Forschungszentrum Jülich, 52425 Jülich, Germany*

[2]*JARA-Fundamentals of Future Information Technology, Jülich-Aachen Research Alliance, Forschungszentrum Jülich and RWTH Aachen University, Germany*

[3]*Department of Physics, University of Warwick, Coventry CV4 7AL, UK*

[4]*RIKEN Center for Emergent Matter Science and Advanced Device Laboratory, 351-0198 Saitama, Japan*

[5]*Peter Grünberg Institut (PGI-10), Forschungszentrum Jülich, 52425 Jülich, Germany*

SUBSTRATE PREPARATION AND NANOWIRE GROWTH

Preparation of the patterned substrate

Here, we provide additional information of the 3-step electron beam (e-beam) lithography fabrication process of the pre-patterned Si substrate for the specially designed selective growth of the InAs NWs. Images obtained with scanning electron microscope (SEM) after the second and third step are presented in Figure S1. An array of $3\,\mu$m wide squares with a pitch of $10\,\mu$m defined in the 20 nm SiO_2 layer covering Si(100) substrate is shown in Figure S1 (a). The patterned SiO_2 layer is used as a mask for etching in the Si(100) substrate 300 nm deep square-shaped troughs with Si(111) facets. Such a trough looks like in Figure S1 (b). The final result of the substrate preparation is illustrated in Figure S1 (c): pairs of 80 nm wide holes are etched in the SiO_2 layer covering adjacent Si(111) facets of a trough. Finally, a focus ion beam (FIB) cross-sectional cut of such a hole on the Si(111) facet is depicted in Figure S1 (d).

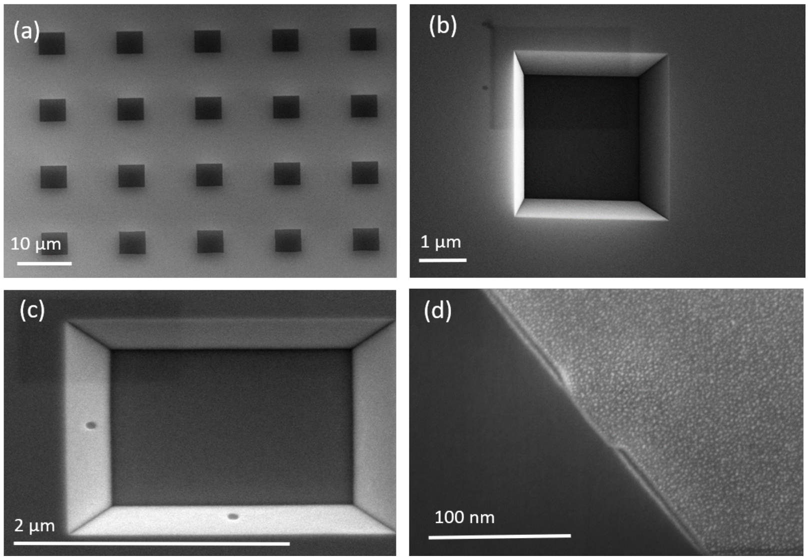

Figure S1. (a) SEM micrograph of 3 μm wide squares etched in the 20 nm SiO_2 layer covering the Si (100) substrate. (b) Square-shaped trough with Si(111) facets. (c) Pair of etched holes in the SiO_2 layer covering Si (111) adjacent facets. (d) FIB cross-sectional cut showing a Si(111) facet of the trough with the hole etched in the oxide; one can distinguish the oxide layer (black) and the Pt metal (grey).

Figure S2 shows a FIB cross-sectional view of a trough covered by the PMMA resist layer before the e-beam lithography of the hole pairs. The Si (111) surface is covered by a SiO_2 layer. One finds that the resist becomes thinner towards the outer edges in comparison to the inner part of the trough and the oxide layer varies on different Si facets,i.e. 25 nm on the Si(111) facet and 18 nm on the Si(100) facet.

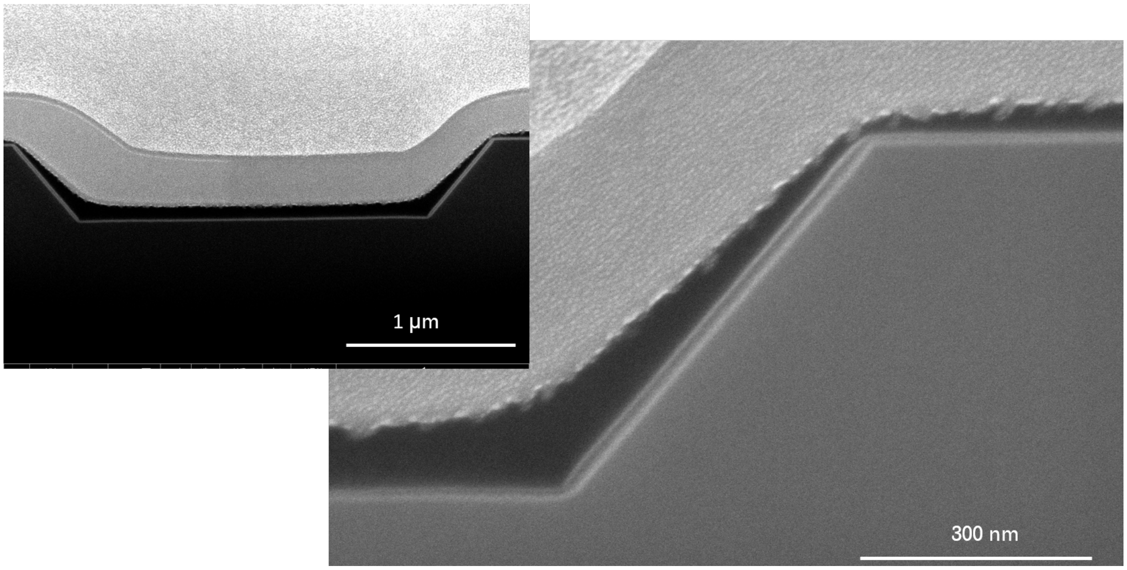

Figure S2. The complete profile of the PMMA resist (black) on top of a trough and the sputtered Pt (grey) used for protection during the FIB cut. Below the resist, one can see the thin SiO_2 layer.

Issues related to the substrate preparation and nanowire growth

During the development of the substrate preparation various issues occurred. Resist sticking problems (cf. Figure S3 (a)) have been tackled by treating the SiO_2 surface with O_2 plasma (power 200 W, flow 300 SCCM) and using HMDS baked at 130°C before spinning the resist. Oxide irregular growth on different Si facets together with not optimised etching parameters are the causes of the parasitic growth shown in Figure S3 (b). By growing a sufficiently thick oxide on Si (100) and Si (111) facets, i.e. 18 nm and 25 nm, respectively and adjusting the RIE and HF etching parameters this problem could be resolved. In order to prevent stunted nanowire growth (cf. Figure S3 (c)), a moderately high As flux of 3.5×10^{-5} torr was used during the nanowire growth. If the dose for e-beam writing is too high, the holes in the SiO_2 layer are getting too large. This results in the growth of two nanowires per hole, as can be seen in Figure S3 (d). To avoid this, the e-beam writing parameters have been varied to find the right doses.

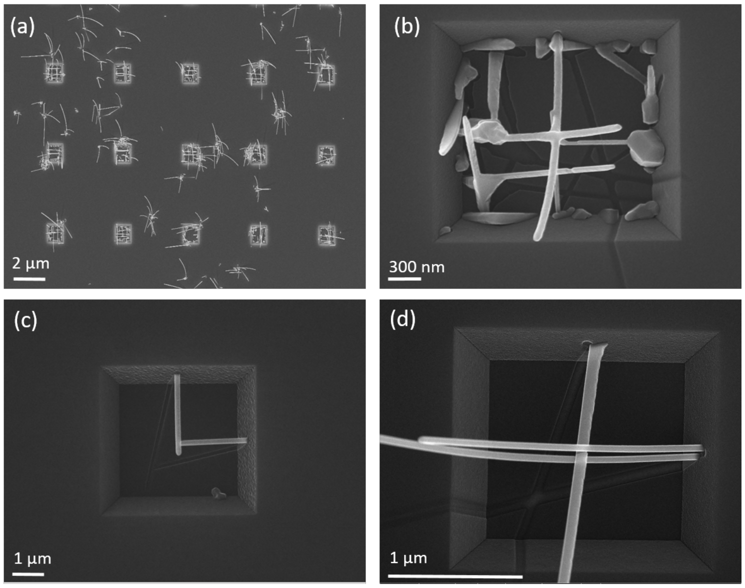

Figure S3. (a) Hard mask (SiO_2) etched away during HF etching caused by insufficient sticking of PMMA to SiO_2 and its tripping. (b) Prasitic growth, nanowires and crytallites, due to inappropiate oxide thickness. (c) Low arsenic fluxes causes stunted nanowire growth. (d) Higher e-beam dose for writing the holes leads to larger holes in the oxide and finally to the growth of two nanowires in one hole.

Randomly grown nanowires

As a proof of concept for growing in-situ Nb/InAs nanowire Josephson junctions, the InAs nanowires were also grown randomly on Si(111) facets. The processing is less elaborated, since it does not require the preparation of holes in the oxide covering Si(111) facets. Instead of thermal oxidation, after processing the troughs, the Si substrate is oxidized using a H_2O_2 solution for 120 sec. The obtained oxide contains pin holes necessary for the nucleation of the nanowires. Since this is an uncontrolled growth process, the nanowire Josephson junction can be obtained if two InAs nanowires grow in the right geometrical configuration to realize the Nb shadow deposition on one of the nanowire (cf. Figure S4).

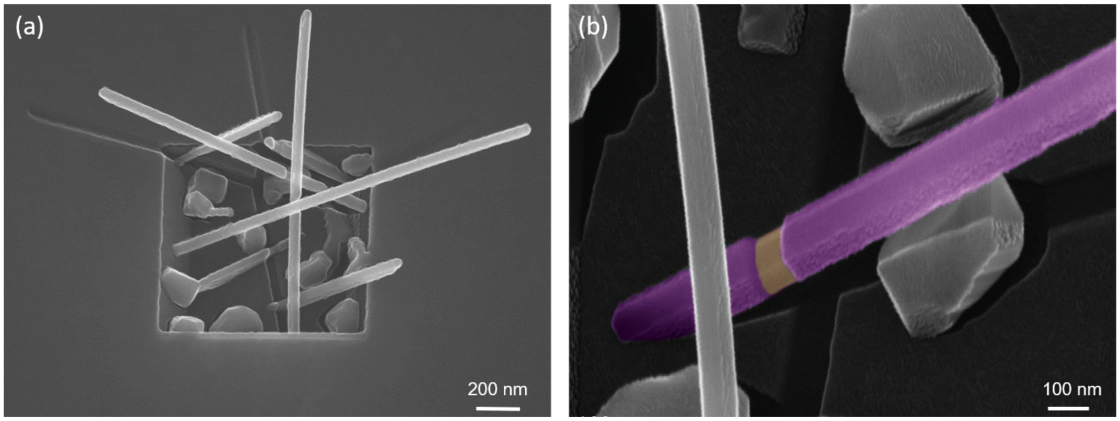

Figure S4. (a) Random nanowire growth on the trough facets and parasitic growth due to larger nucleation sites. (b) Detail showing randomly grown nanowires crossing each other at a small distance. As a consequence, the Nb shell (pink) on the lower nanowire present a gap due to the shadow of the nanowire above during the metal deposition and a nanowire Josephson junction is obtained.

TRANSMISSION ELECTRON MICROSCOPY

As seen in Figure S5 (d) and similar to the previous observation related to Al half-shells [S3], a single Nb grain (which is larger than $\sim$15 nm) can grow beyond the length of a typical polytypic region, assuming that the amorphisation takes place later through solid diffusion (also note that the crystal phases of InAs is not visible in its $<1\bar{1}00>$ zone axis, which was used to image the Nb layer more clearly. Hence, a comparison with the image in Figure S5 (a), which is imaged in the $<11\bar{2}0>$ zone axis and typical of defect density has to be made, in order to gauge polytypic segment lengths). This extended growth of Nb beyond the polytypic segment size could be due to the nucleation-and-expansion type growth process of metal grains or Nb growth being driven by factors other than those related to surface and interface [S4]. However, the possibility of the rough micro-facets formed by ZB inclusions leading to more nucleations (and hence smaller grain size) cannot be discounted [S5].

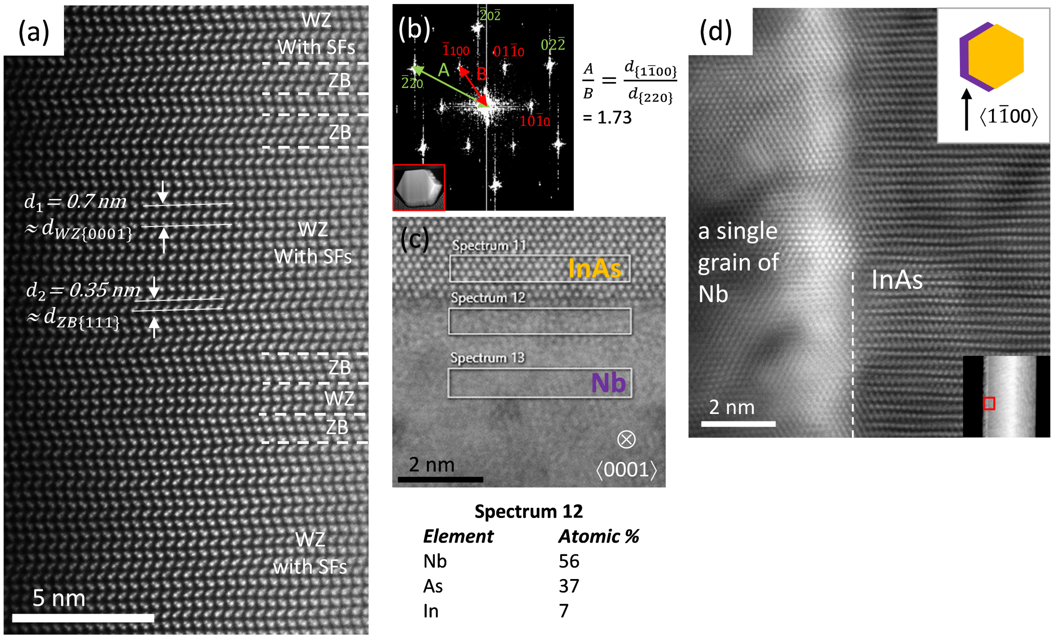

Figure S5. (a) Annular dark field (ADF) STEM image of a nanowire grown using the selective growth technique, showing the polytypic crystal structure. (b) FFT of the cross-section shown in Figure 2(c) in the main manuscript, showing contributions from both polytypes and confirming the $\{11\bar{2}0\}$ ($\{110\}$ ZB equivalent) type side facets. The indices and the ratio between the lattice spacings are also given [S1, S2]. (c) Method of ascertaining composition of the ~1 nm thin amorphous layer, along with an example of the values extracted. (d) ADF STEM image of a large Nb grain oriented such that [110]Nb||[0001]InAs in the axial direction. The red square in the bottom right inset indicates the area of acquisition and the top right inset shows a schematic indicating projections in the $<1\bar{1}00>$ viewing direction. The white broken line indicates the $\{11\bar{2}0\}$ nanowire facet edge. However, note that part of the Nb shell is still viewed on the nanowire side along the projection, due to the half-shell growth on multiple facets around the nanowire as shown by the inset schematic.

DIFFERENTIAL RESISTANCE AND SUBGAP FEATURES

In Figure S6 the differential resistance is shown as function of the voltage across the junction for a gate voltage of $V_g = 7\,V$. For voltages below 0.3 mV, multiple sub-gap features are visible which can be attributed to multiple Andreev reflections. Additionally, a double peak feature is observable around 0.7 mV. The latter could be either a signature of multiple Andreev reflections, too, or the superconducting gaps of the Nb shell, the NbTi contacts or

some kind of hybridized electronic mixture of both materials. Owing to the low junction resistance in the normal state, we did not bias with currents resulting in a voltage drop corresponding to twice the gap of Nb because the large current would burn the device. Thus, we did not reach a range in the differential resistance which would allow us to extract the gap energy of Nb.

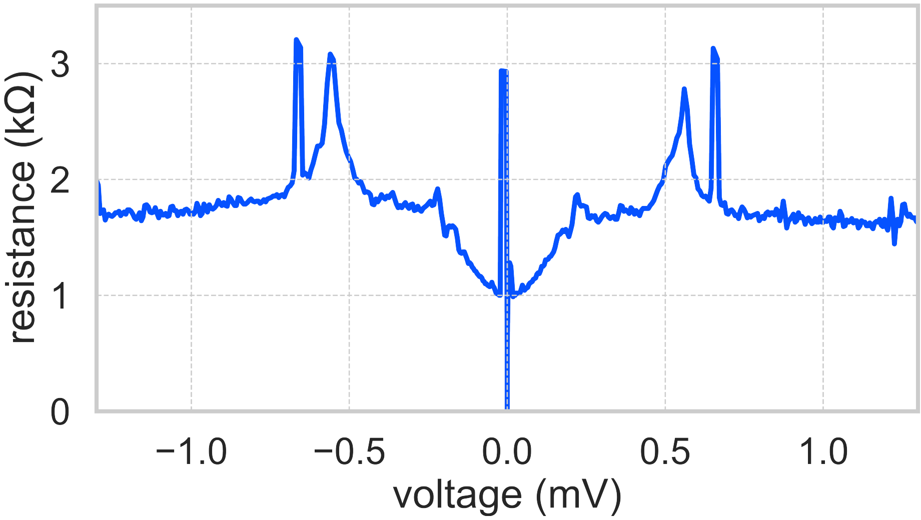

Figure S6. Voltage-dependent differential resistance trace for a gate voltage of $V_g = 7\,V$.

[S1] D. Kriegner, C. Panse, B. Mandl, K. A. Dick, M. Keplinger, J. M. Persson, P. Caroff, D. Ercolani, L. Sorba, F. Bechstedt, J. Stangl, and G. Bauer, Unit cell structure of crystal polytypes in InAs and InSb nanowires, Nano Letters **11**, 1483 (2011).

[S2] http://www.ioffe.ru/sva/nsm/semicond/.

[S3] P. Zellekens, R. Deacon, P. Perla, H. A. Fonseka, T. Mörstedt, S. A. Hindmarsh, B. Bennemann, F. Lentz, M. I. Lepsa, A. M. Sanchez, D. Grützmacher, K. Ishibashi, and T. Schäpers, Hard-gap spectroscopy in a self-defined mesoscopic InAs/Al nanowire Josephson junction, Phys. Rev. Applied **14**, 054019 (2020).

[S4] T. Kanne, M. Marnauza, D. Olsteins, D. J. Carrad, J. E. Sestoft, J. de Bruijckere, L. Zeng, E. Johnson, E. Olsson, K. Grove-Rasmussen, and J. Nygård, Epitaxial Pb on InAs nanowires, arXiv preprint arXiv:2002.11641 (2020), arXiv:2002.11641 [cond-mat.mes-hall].

[S5] S. G. Ghalamestani, M. Heurlin, L.-E. Wernersson, S. Lehmann, and K. A. Dick, Growth of InAs/InP core–shell nanowires with various pure crystal structures, Nanotechnology **23**, 285601 (2012).
